# A Novel Thrombosis-Related Signature for Predicting Survival and Drug Compounds in Glioblastoma

**DOI:** 10.1155/2022/6792850

**Published:** 2022-07-13

**Authors:** Wen-Jing Zeng, Yu-Fang Cao, He Li, Zhi-Cheng Gong, Wantao Wu, Peng Luo, Jian Zhang, Zaoqu Liu, Hao Zhang, Quan Cheng

**Affiliations:** ^1^Department of Pharmacy, Xiangya Hospital, Central South University, Changsha, Hunan, China; ^2^National Clinical Research Center for Geriatric Disorders, Xiangya Hospital, Central South University, Changsha, Hunan, China; ^3^Department of Pharmacy, Hunan University of Medicine, Huaihua, Hunan, China; ^4^Hunan Clinical Research Center in Gynecologic Cancer, Hunan Cancer Hospital, The Affiliated Cancer Hospital of Xiangya School of Medicine, Central South University, Changsha, Hunan, China; ^5^Department of Oncology, Xiangya Hospital, Central South University, Changsha, Hunan, China; ^6^Department of Oncology, Zhujiang Hospital, Southern Medical University, Guangzhou, China; ^7^Department of Interventional Radiology, The First Affiliated Hospital of Zhengzhou, Zhengzhou, Henan, China; ^8^Department of Neurosurgery, Xiangya Hospital, Central South University, Changsha, Hunan, China

## Abstract

Glioblastoma is the most common primary tumor in the central nervous system, and thrombosis-associated genes are related to its occurrence and progression. Univariate Cox and LASSO regression analysis were utilized to develop a new prognostic signature based on thrombosis-associated genes. Gene ontology (GO), Kyoto Encyclopedia of Genes and Genomes (KEGG), and HALLMARK were used for functional annotation of risk signature. ESTIMATE, MCP-counter, xCell, and TIMER algorithms were used to quantify immune infiltration in the tumor microenvironment. Genomics of Drug Sensitivity in Cancer (GDSC) was used for selecting potential drug compounds. Risk signature based on thrombosis-associated genes shows moderate performance in prognosis prediction. The functional annotation of the risk signature indicates that the signaling pathways related to the cell cycle, apoptosis, tumorigenesis, and immune suppression are rich in the high-risk group. Somatic mutation analysis shows that tumor-suppressive gene *TP53* and oncogene *PTEN* have higher expression in low-risk and high-risk groups, respectively. Potential drug compounds are explored in risk score groups and show higher AUC values in the low-risk score group. A nomogram with valuable prognostic factors exhibits high sensitivity in predicting the survival outcome of GBM patients. Our research screens out multiple thromboses-associated genes with remarkable clinical significance in GBM and further develops a meaningful prognostic risk signature predicting drug sensitivity and survival outcome.

## 1. Introduction

Glioma is the most common primary malignancy in the central nervous system, accounting for approximately 80% of primary malignant brain tumors in adults [1]. According to the 2016 World Health Organization (WHO) category of the Central Nervous System Tumors, glioma can be classified into astrocytoma, oligodendroglioma, oligodendrocyte, and glioblastoma (GBM), including WHO grades I-IV based on its malignancy degree [2]. The overall survival of GBM patients ranges from 1 to 15 years, with great individual differences [3]. At present, the treatment of GBM is mainly based on maximum resection, adjuvant radiotherapy, and TMZ chemotherapy [4, 5]. Due to the highly invasive nature of GBM, it is difficult to completely remove the tumor by neurosurgery [5]. The residual lesions are prone to resistance to radiotherapy and chemotherapy, thus leading to recurrence and malignant progression of GBM. With the development and application of sequencing technology and molecular diagnostic technology, a more objective and accurate tumor classification system has been established clinically at present [6, 7]. However, currently, known molecular markers can only partially explain the prognosis of GBM patients.

Venous thromboembolism (VTE), which comprises deep vein thrombosis (DVT) and pulmonary embolism (PE), occurs extensively in various cancers, including GBM. The incidence of VTE in GBM is high, and complications are easy to occur. VTE has been found in 17% of patients with GBM, and the cumulative probability of VTE was 14.3% at 6 months and 16.8% after 12 months [8]. In addition to VTE, the intratumoral thrombosis in GBMs, which may cause ischemia or necrosis due to vascular obstruction, is also an essential factor affecting the prognosis of GBM patients. Risk factors for GBM-associated thrombosis include general clinical characteristics, such as advanced age, grade, tumor size, reduced activity, and thrombosis-associated biomarkers or genes. Recent studies have shown that the expressions of many thrombosis-associated proteins in GBMs, such as tissue factor (TF), human epidermal growth factor (VEGF), and D-dimer, are correlated with pathological tumor grade and poor prognosis [8, 9]. TF is a transmembrane glycoprotein that plays a vital role in thrombosis, and its expression is significantly upregulated in GBM [10, 11]. In addition, *PTEN* loss and hypoxia can further upregulate TF expression, thereby inducing angiogenesis and local necrosis of glioblastomas [12]. Previous studies indicated that thrombosis-associated genes mediated GBM metastasis, thrombosis, and other biological processes, suggesting that thrombosis-associated genes changes may play an essential role in diagnosis and prognostic prediction of glioblastomas [13].

This study aims to identify prognostic thrombosis-associated gene signatures by integrating the transcriptional data of GBM in The Cancer Genome Atlas (TCGA) and the Chinese GBM Genome Atlas (CGGA) databases. We constructed an independent prognostic risk score model based on the mRNA expression of 13 thrombosis-associated genes that were phenotypically significantly associated with immune invasion and somatic mutation in GBM using univariate Cox and LASSO regression analysis. We believe that the findings may provide a theoretical basis for the molecular diagnosis and individualized treatment for GBMs.

## 2. Methods

### 2.1. Datasets Source

Gene expression profiles and corresponding clinical information were downloaded from the TCGA database (https://xena.ucsc.edu) and the CGGA database (https://www.cgga.org.cn). Thrombosis-associated genes were obtained from the literature, and the gene list is shown in Table S1. GBM samples with overall survival (OS) of less than 30 days in TCGA and CGGA databases were excluded. The TCGA GBM dataset is randomly divided into the training set (train) and the validation set (test). The total TCGA GBM dataset (sum) and CGGA GBM cohort are the two validation sets in this study.

### 2.2. Constitution and Validation of the Prognostic Risk Score Model

First, a univariate Cox proportional hazard regression analysis was performed using the “survival” package in R to identify the thrombosis-associated genes involved in prognosis. Genes with *P* value <0.05 were considered to have significant prognostic potential. The least absolute shrinkage and selection operator (LASSO) regression was performed to further screen the genes with independent prognostic value [14]. Based on the highest *λ* value selected through 1,000 cross-validations in the LASSO method, a set of prognosis genes and their LASSO coefficients (*β*) were obtained [15]. Then, a gene-based survival risk assessment model was established with LASSO coefficients: risk score=∑_*i*=1_^*N*^(Expi × Coei), where *N* = 13, Exp^*i*^ is the expression value of every 13 thrombosis associated genes, and Coe^*i*^ is the corresponding LASSO regression coeﬃcient. Patients in the TCGA and CGGA datasets were divided into high- and low-risk groups according to the median risk score.

### 2.3. Functional Enrichment Analysis

To evaluate the biological pathway associated with the 13 thrombosis-associated genes, the “GSVA” package was used for gene set variation analysis (GSVA), including Gene Ontology (GO), Kyoto Encyclopedia of Genes and Genomes (KEGG), and HALLMARK.

### 2.4. Immunological Function Analysis

According to the expression characteristics of thrombosis genes, cell components or cellular immune responses in the high-risk and low-risk groups were evaluated by the ESTIMATE [16], MCP-counter [17], xCell [18], and TIMER [19] algorithms. Heatmaps displayed the immune responses generated under different algorithms.

### 2.5. Somatic Mutation Analysis

Somatic mutations, including the SNVs, SNPs, and INDELs, were detected using the TCGA mutation data of both the high-risk group (*n* = 146) and low-risk group (*n* = 147) with the R package “maftools” [20]. Fisher's exact test was used to determine the pattern of differential mutation. Genes with a *P* value <0.05 were defined as differentially mutated genes. The cooccurrence and mutually exclusive mutations were identified using the CoMEt algorithm [21]. Somatic mutation visualizations were generated using the R package “maftools.”

### 2.6. Prediction of Chemotherapy Response

According to the public pharmacogenomic databases, PRISM Repurposing dataset (PRISM, https://depmap.org/portal/prism/) and Cancer Therapeutics Response Portal (CTRP, https://portals.broadinstitute.org/ctrp), and according to a previous study, drug sensitivity (IC50) values were predicted by the R package “pRRophetic” [22].

### 2.7. Prognostic Model Based on Clinical Features and Risk Score

Univariate Cox and Multivariate Cox proportional hazard regression analysis was performed to identify independent prognostic risk factors, including the risk score and clinical characteristics (age, gender, subtype, *IDH* mutation, chemotherapy, and radiotherapy) using the “survival” package. The independent prognostic factors were then used to construct a nomogram chart and a calibration curve to evaluate and compare the predicted and actual OS probabilities for GBM patients at 1, 2, and 3 years. The nomogram chart and the calibration curves were constructed using the R package “RMS.”

### 2.8. Statistical Analysis

All statistical analyses were performed using SPSS 22.0 or R software. The normality of variables was tested using the Shapiro-Wilk normality test. For normally distributed variables, significant quantitative differences between and among groups were determined by a two‐tailed *t*-test or one‐way ANOVA, respectively. For nonnormally distributed variables, significant quantitative differences between and among groups were determined by a Wilcoxon test or a Kruskal-Wallis test, respectively. The chi-square test was used to analyze the correlation of the classified data. Kaplan-Meier survival curve and log-rank tests were used to detect the prognostic difference in different groups. The R package “survivalROC” was used to plot time-dependent receiver operating characteristic (ROC) curves and calculate the area under the curve (AUC) [23]. *P* values <0.05 were considered statistically significant.

## 3. Results

### 3.1. Identification and Verification of Prognostic Risk Score Model Based on Thrombosis-Associated Gene Signature in GBM

**Figure 1:**
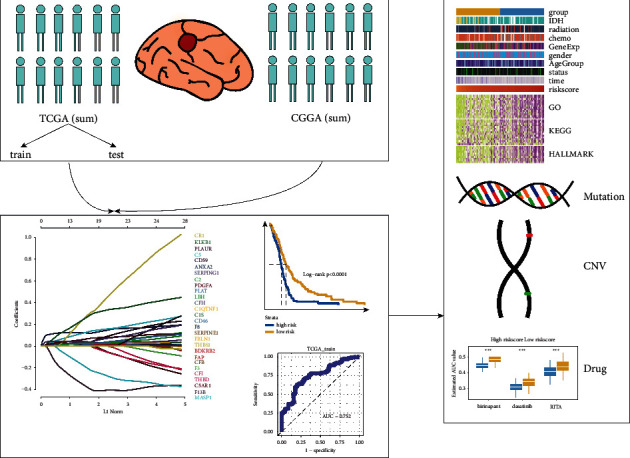
Flow diagram of this study.

**Figure 2:**
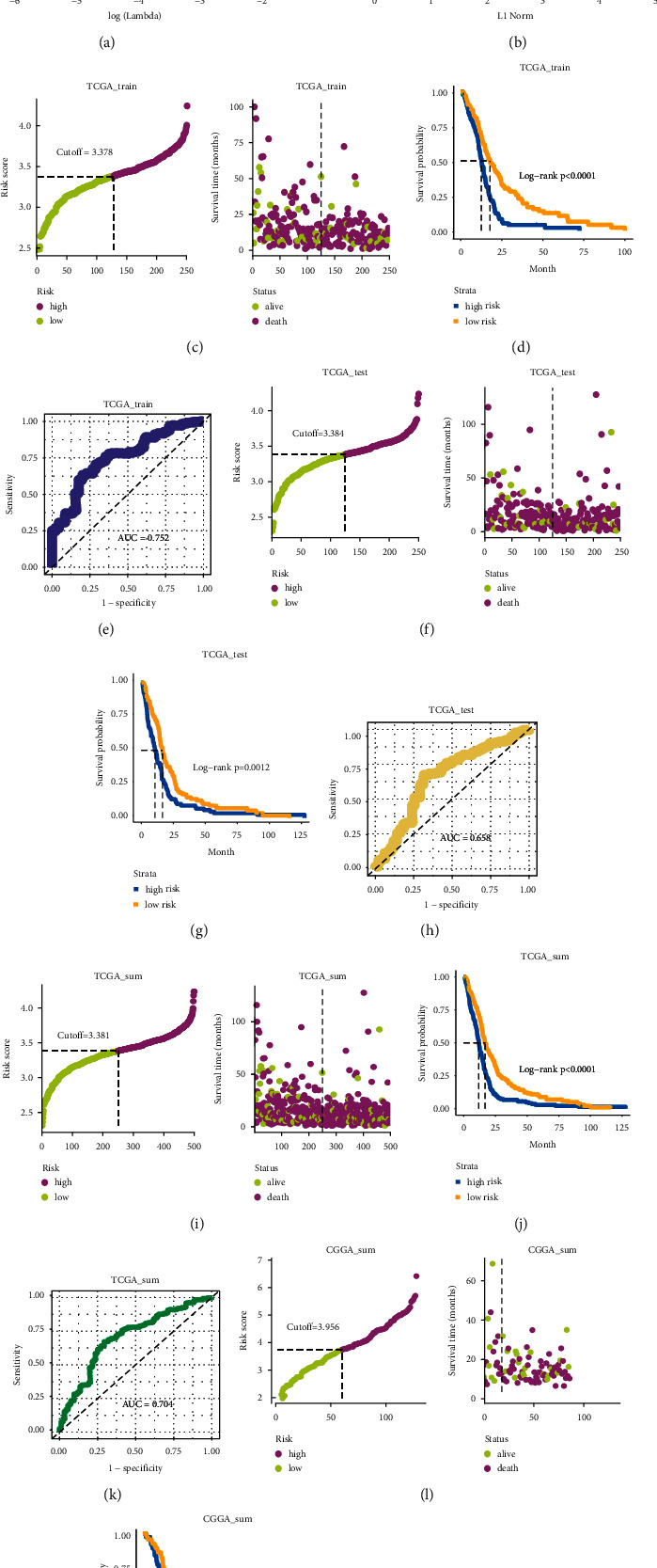
Identification and verification of prognostic risk score model based on thrombosis-associated gene signature in glioblastomas. (a) 1000 cross-validations were used for tuning parameters selection in the LASSO regression model. (b) LASSO coefficients profiles of 13 prognostic thrombosis-associated genes. (c) The patients' risk scores and survival status in the TCGA training set. (d) Kaplan-Meier survival curves for OS of patients between high-risk and low-risk groups in TCGA training cohort. (e) ROC curve analysis for predicting survival in TCGA training cohort. (f) The distribution of patients' risk scores and survival status in the TCGA test cohort. (g) Kaplan-Meier survival curves for OS of glioblastoma patients between high-risk and low-risk groups in TCGA test cohort. (h) ROC curve analysis for predicting survival in TCGA test cohort. (i) The distribution of patients' risk scores and survival status in the TCGA sum cohort. (j) Kaplan-Meier survival curves for OS of glioblastoma patients between high-risk and low-risk groups in TCGA sum cohort. (k) ROC curve analysis for predicting survival in TCGA sum cohort. (l) The distribution of patients' risk scores and survival status in CGGA cohort. (m) Kaplan-Meier survival curves for OS of glioblastoma patients between high-risk and low-risk groups in CGGA cohort. (n) ROC curve analysis of risk score based on thrombosis-associated genes in CGGA cohort.

The overall study design is shown in Figure 1. In total, 140 thrombosis-associated genes were identified from the literature, of which 124 genes expressed in both TCGA and CGGA datasets were chosen for subsequent analyses. To identify prognostic thrombosis-associate genes in GBM, LASSO regression analysis was performed using TCGA training, and 13 genes were screened out of 124 genes (Figures 2(a) and 2(b)). The LASSO regression coefficients of 13 thrombosis-associated genes are shown in Table S2. Then, a risk score model was established based on thrombosis-associated gene expression with LASSO coefficients: risk score = (0.1235∗*ANXA2* mRNA expression + 0.1175∗*C5* mRNA expression +0.0195∗*CD59* mRNA expression + 0.0309∗*CFH* mRNA expression + 0.2343∗*CR1* mRNA expression – 0.3472∗*F13B* mRNA expression + 0.0079∗*FAP* mRNA expression + 0.2369∗*KLKB1* mRNA expression + 0.0247∗*LBH* mRNA expression + 0.0602∗*PDGFA* mRNA expression + 0.0104∗*PLAT* mRNA expression + 0.0419∗*SERPING1* mRNA expression – 0.1703∗*MASP1* mRNA expression). According to the median risk score, GBM patients in the TCGA training set were divided into high-risk and low-risk groups. The risk score distribution and survival status of patients are shown in Figure 2(c). Kaplan-Meier survival curves indicated that high-risk patients' OS was significantly worse than that of low-risk patients (Figure 2(d)). A time-dependent ROC curve analysis evaluated the risk score model's predictive accuracy. The result showed that the AUC was 0.752, which proved that the risk score model had good accuracy and predictive ability within the TCGA training cohort (Figure 2(e)). Subsequently, the TCGA test set and TCGA sum set were used to verify the predictive prognosis ability of the risk score model. The risk score distribution and survival status of patients in the TCGA test set and TCGA sum set are shown in Figures 2(f) and 2(i). We also found that the OS of GBM patients in the high-risk group was significantly shorter than that in the low-risk group in the TCGA test set and TCGA sum set (Figures 2(g) and 2(j)). The AUC of the ROC curve in the TCGA test set and sum set were 0.658 and 0.704, respectively (Figures 2(h) and 2(k)).

Further verification in the CGGA dataset showed similar tendencies that the GBM patients in the high-risk group have a poorer prognosis compared to those in the low-risk group (Figure 2(m)). The AUC of the ROC curve was 0.714 (Figure 2(n)). The risk score distribution and survival status of patients in the CGGA dataset are shown in Figure 2(l). These findings revealed that the risk score model based on thrombosis-associated signature has an excellent survival predicting power of patients' OS.

Additionally, we detected the expression of 13 thrombosis-associated genes in the TCGA and CGGA datasets. Heatmaps of 13 thrombosis-associated genes are shown in Figure S1, in which *F13B* and *MASP1* were regarded as protective genes for expression level decreases with risk score gradually increases, and others were risky genes for growing together. To assess the association between 13 thrombosis-associated genes' expression and patients' OS, patients were divided into high-expression and low-expression groups based on the median of gene expression, and Kaplan-Meier curves were performed. As shown in Figure S2, the increased expression of *MASP1* and *F13B* indicated an excellent survival in GBM patients in the TCGA dataset. In contrast, the high expressions of *ANXA2*, *C5*, *CD59*, *CFH*, *CR1*, *FAP*, *KLKB1*, *LBH*, and *PDGFA* predicted a poor prognosis in GBM patients. The association between *IDH* mutation status and the 13 thrombosis-associated genes was explored in the TCGA and CGGA datasets. *ANXA2*, *C5*, *CFH*, *FAP*, *PDGFA*, *PLAT*, and *SERPING1* had higher expressions in the *IDH* WT tumors in the TCGA and CGGA datasets, which predicted worse survival (Figures S3A and S3B).

### 3.2. Functional Enrichment Analyses for Thrombosis-Associated Gene Signature

**Figure 3:**
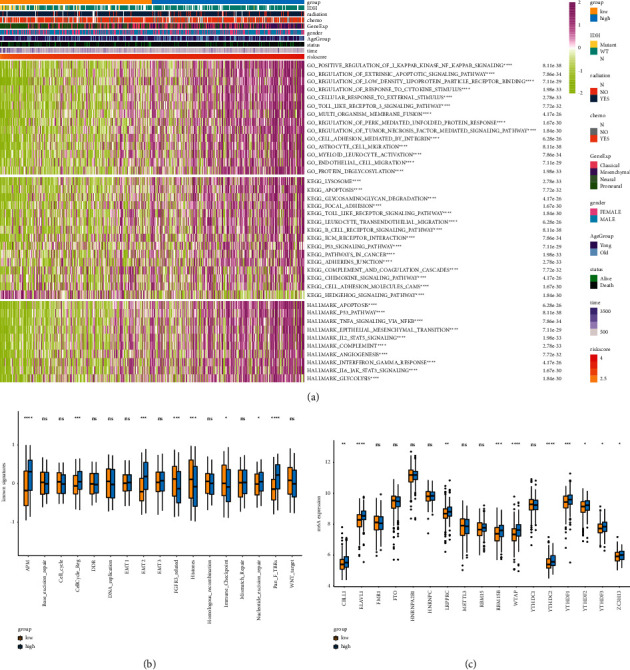
Functional enrichment analyses of a thrombosis-associated gene signature in glioblastomas. (a) GO, KEGG, and HALLMARK analyses for thrombosis-associated prognostic genes in TCGA. (b) The expression of known signatures in high-risk and low-risk groups in TCGA. (c) The expression of m6A-related genes in high-risk and low-risk groups in TCGA. GO, Gene Ontology; KEGG, Kyoto Encyclopedia of Genes and Genomes; m6A, N6-methyladenosine.

To explore the potential biological function of a prognostic thrombosis-associated gene signature in GBM, GSVA was performed. As shown in Figure 3(a), GO analysis results showed that the thrombosis-associated gene signature was mainly enriched in the positive regulation of Kappa B kinase NF-*κ*B signaling, the regulation of extrinsic apoptotic signaling pathway, the regulation of low-density lipoprotein particle receptor binding, the regulation of response to cytokine stimulus, and so forth. KEGG analysis results indicated that the thrombosis-associated gene signature was primarily concentrated in the lysosome, the apoptosis, the glycosaminoglycan degradation, the focal adhesion, the Toll-like receptor signaling pathway, and so forth (Figure 3(a)). In addition, the HALLMARK analysis results displayed that the thrombosis-associated gene signature was also principally enriched in the apoptosis pathway, the P53 pathway, the TNF*α* signaling via NF-*κ*B, the epithelial-mesenchymal transition pathway, and so forth (Figure 3(a)).

In addition, to investigate the correlation between the expression of thrombosis-associated gene signature and the known signature, GBM patients in the TCGA dataset were divided into high-risk and low-risk groups based on the median risk score. We found significant differences in APM expression, cell cycle regulation, EMT2, FGFR3 related signature, histones, immune checkpoint, nucleotide excision repair, and Pan_F_TBRs signature between the low-risk group and the high-risk group (Figure 3(b)). Differential expression analysis of m6A-related genes between the low-risk group and the high-risk group showed that the expressions of *CBLL1*, *ELAVL1*, *LRPPRC*, *RBM15B*, *WTAP*, *YTHDC2*, *YTHDF1*, *YTHDF2*, *YTHDF3*, and *ZC3H13* in the high-risk group were significantly higher than those in the low-risk group in the TCGA GBM dataset (Figure 3(c)). Notably, the expression of *WTAP* in the high-risk group was also significantly higher than that in the low-risk group in the CGGA dataset (Figure S4).

### 3.3. Immunological Function Analysis for Prognostic Thrombosis-Associated Gene Signature

**Figure 4:**
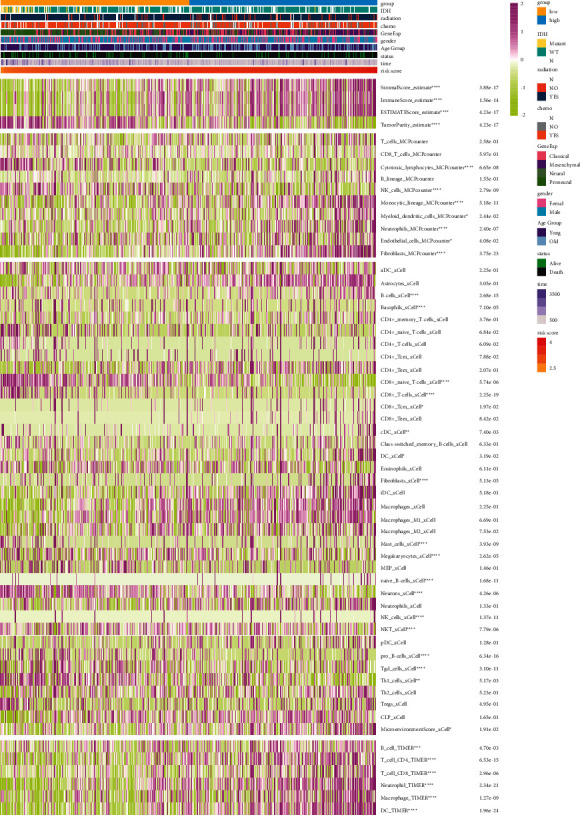
Immunological function analysis of thrombosis-associated gene signature in glioblastomas. Heatmap shows the differential cellular immune responses between high-risk and low-risk groups analyzed by ESTIMATE, MCP-counter, TIMER algorithms, and xCell in TCGA.

To verify the relationship between prognostic thrombosis-associate gene signature and immune responses, we analyzed the correlation between risk score and immune factors in the TCGA GBM dataset based on ESTIMATE, MCP-counter, TIMER, and xCell. As shown in Figure 4, the risk score showed a negative correlation with Tumor Purity and a positive correlation with Stromal Score, Immune Score, and ESTIMATE Score. Moreover, correlation analysis between risk score and immune cell subpopulations based on the MCP-counter revealed that risk score was negatively correlated with cytotoxic lymphocytes and NK cells but positively associated with monocytic lineage, myeloid dendritic cells, neutrophils, endothelial cells, and fibroblasts (Figure 4). Additionally, correlation analysis between risk score and immune cell subpopulations based on TIMER (Figure 4) and xCell (Figure 4) also indicated that risk score has a correlation with B cells, CD8+ cells, CD4+ cells, neutrophil, macrophage, and dendritic cells (DC). Similar results were obtained in the CGGA dataset (Figure S5).

### 3.4. Somatic Mutation Analysis for Prognostic Thrombosis-Associated Gene Signature

**Figure 5:**
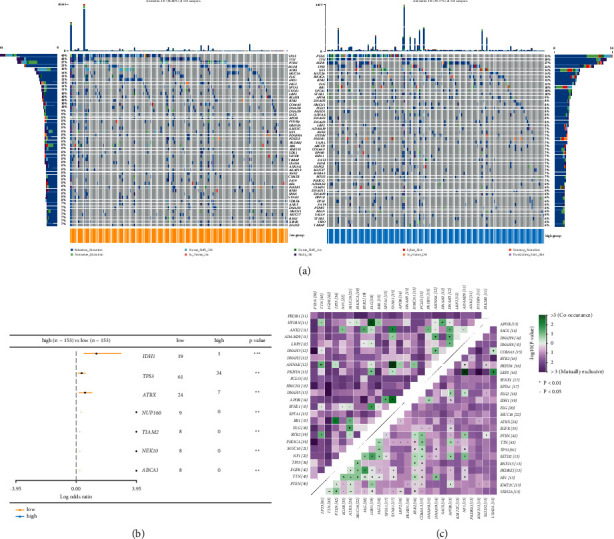
Somatic mutation analysis of thrombosis-associated gene signature in glioblastomas. (a) The waterfall plot displays the mutation distribution of the top 50 most frequently mutated genes. (b) The Forest plot illustrates the top 7 most significantly differentially mutated genes between high-risk and low-risk groups. (c) The heatmap shows the mutually cooccurring and exclusive mutation genes. The color and symbol in each cell represent the statistical significance of the exclusivity or cooccurrence for each pair of genes.

To identify differences in somatic mutation between the high-risk and low-risk groups, we analyzed the somatic mutation in the TCGA GBM dataset. In the low-risk group, 16 genes were mutated in more than 10% of the samples, while only 12 genes met the criteria in the high-risk group, of which 9 genes overlapped (Figure 5(a)). The top 50 with the highest mutation frequency in the low-risk and high-risk groups are shown in Figure 5(a). Interestingly, *TP53* [24], *TTN* [25], *PTEN* [26], and *EGFR* [27] occupied the top four positions in both low-risk and high-risk groups, and they are interacting with each other to regulate various biological processes related to GBM, suggesting that they may be involved in tumor deterioration. In addition, currently recognized GBM prognostic genes (*IDH1*, *TP53*, *ATRX*, *NUP16*, *TIAM2*, *NEK10*, and *ABCA1*) showed significant differences in mutation frequency between the high-risk and low-risk groups (Figure 5(b)). Next, we studied cooccurrence and exclusive mutations in the 25 most common mutated genes using the CoMEt algorithm. Except for the prevailing mutually exclusive mutation landscape, there were three unique gene pairs. Two genes exhibited cooccurrence, including *HYDIN* and *FLG*, *AHNAK* and *SYNE1*, and *NF1* and *LRP2* (Figure 5(c)), suggesting their probable complementary effect in the same pathway. More interestingly, some genes had differential mutation frequencies between the two groups.

### 3.5. Identification of Potential Therapeutic Agents for High-Risk Score Patients

**Figure 6:**
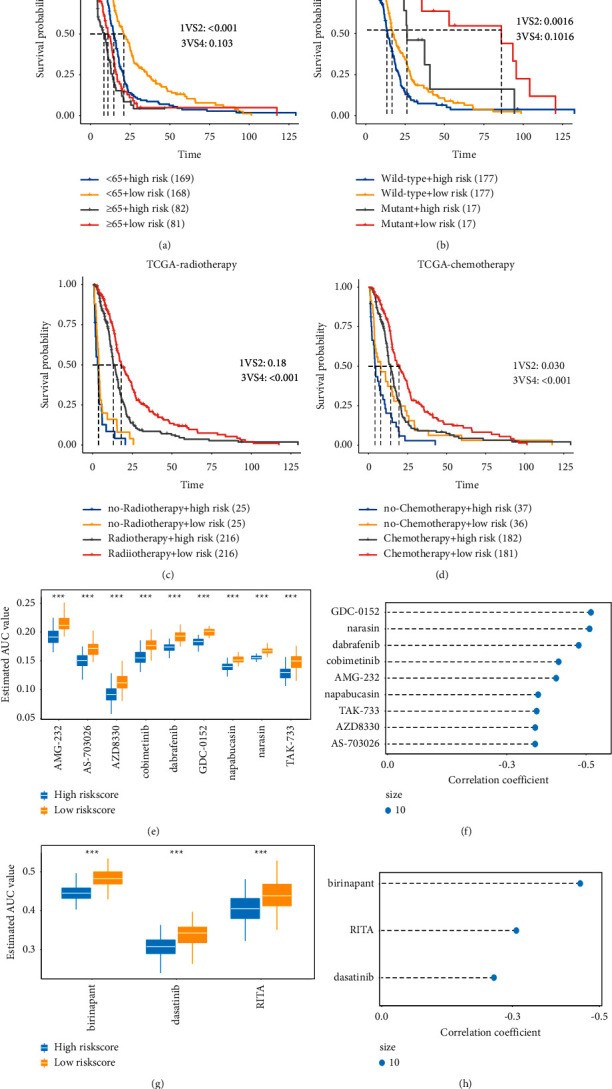
Survival analysis of high-risk and low-risk groups with different clinicopathological factors and the prediction of chemotherapy response. (a) Kaplan-Meier survival curves of patients in the high-risk and low-risk groups aged 65 years or older and those below 65 years of age in the TCGA glioblastoma cohort. (b) Kaplan-Meier survival curves of patients in the high-risk and low-risk group with *IDH* mutation or wild-type *IDH* in TCGA glioblastoma cohort. (c, d) Kaplan-Meier survival of patients in the high-risk and low-risk groups receiving radiotherapy and chemotherapy in the TCGA glioblastoma cohort. (e) Box plot depicting the AUC differences of 9 PRISM-derived compounds in risk score groups. (f) Spearman's correlation analysis and differential drug response analysis of 9 PRISM-derived compounds. (g) Box plot depicting the AUC differences of 3 CTRP-derived compounds in risk score groups. (h) Spearman's correlation analysis and differential drug response analysis of 3 CTRP-derived compounds.

To determine potential therapeutic agents for GBM patients with a high-risk score, PRISM and CTRP-derived drug response data were analyzed. We first screened candidate drugs that responded differently between the high-risk and low-risk groups to identify compounds with higher drug sensitivity in GBM patients with a high-risk score. The candidate drug meets two criteria: (1) Drugs respond differently between high-risk and low-risk groups to identify highly drug-sensitive compounds in high-risk GBM patients. (2) Spearman correlation analysis between the estimated AUC values of a candidate drug and risk score was used to select compounds with negative correlation coefficient (Spearman's *r* < −0.25 for CTRR or Spearman's *r* < −0.35 for PRISM). These analyses yielded nine PRISM-derived compounds (AGM-232, AS-703026, AZD8330, Cobimetinib, Dabrafenib, GDC-0152, Napabucasin, Narasin, and TAK-733) (Figures 6(e) and 6(f)) and three CTRP-derived compounds (Birinapant, Dasatinib, and RITA) (Figures 6(g) and 6(h)). All these compounds had lower estimated AUC values in a high-risk group and a negative correlation with risk score (Figures 6(e)–6(h)).

### 3.6. Clinical and Molecular Features of Low-Risk and High-Risk GBM Patients

We performed risk stratification analysis on various clinicopathological features (age, *IDH* mutation, radiotherapy, and chemotherapy) with different groups to evaluate the prognostic value of the risk score model. Older age is a known risk factor for malignant glioblastomas. As shown in Figure 6(a), both GBM patients below 65 years of age and GBM patients aged 65 years or older with a high-risk score had a worse prognosis than those with a low-risk score in the TCGA GBM cohort. A similar result was obtained in the CGGA dataset (Figure S6A). *IDH* mutation is currently recognized as a molecular marker for prognostic prediction of GBM patients [11, 13, 20]. To determine whether the risk score can be used as a prognostic indicator independent of *IDH* mutation, we performed a stratified analysis of patients in the high-risk and low-risk groups based on *IDH* mutation status. In the TCGA GBM and CGGA datasets, *IDH* wild-type patients with high-risk scores had a worse prognosis than those with a low-risk score, and the same trend was also found in *IDH* mutation patients (Figures 6(b) and S6B). To investigate the risk score based on thrombosis-associated signature concerning prognosis among GBM patients with radiotherapy and chemotherapy, Figures 6(c) and 6(d) show that patients in the high-risk group without radiotherapy or chemotherapy indicated the worst prognosis. In contrast, patients in the low-risk group with radiotherapy or chemotherapy showed the best forecast. Similar results were obtained in the CGGA dataset (Figures S6C and S6D). The above results proved that the risk score model based on thrombosis-associated genes could serve as an independent prognostic factor for GBM patients.

### 3.7. Construction and Evaluation of the Clinical-Featured Risk Model of Glioblastoma

**Figure 7:**
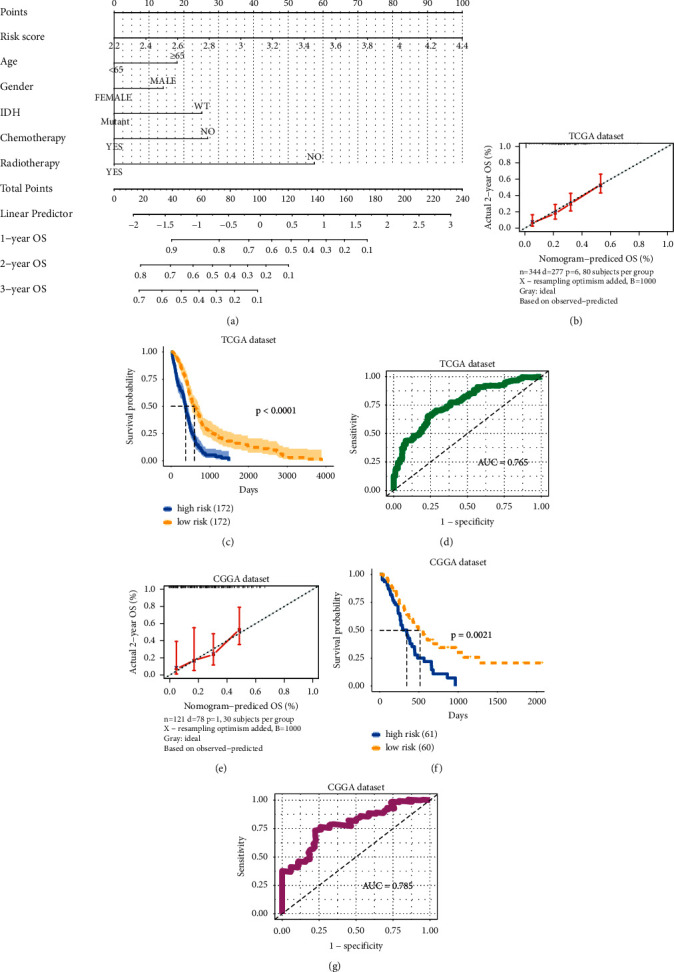
Establishment and assessment of the clinical-risk model. (a) Prognostic nomogram for glioblastomas containing age, gender, *IDH* mutation status, chemotherapy, radiotherapy, and risk score. (b) 2-Year calibration curve of nomogram in TCGA glioblastoma cohort. (c) Kaplan-Meier survival for OS based on the score of the clinical-risk model in the TCGA glioblastoma cohort. (d) ROC curves for the clinical-risk model in TCGA glioblastoma cohort. (e) Calibration curves for 2-year OS in CGGA cohort. (f) Kaplan-Meier survival for OS based on the score of the clinical-risk model in the CGGA cohort. (g) ROC curves for the clinical-risk model in the CGGA cohort.

Then, we built a nomogram to predict the 1-year, 2-year, and 3-year overall survival with the risk score based on thrombosis-associated signature and clinical factors (age, gender, *IDH* mutation status, radiotherapy, and chemotherapy) (Figure 7(a)). The calibration chart showed that the nomogram performed well at predicting the 1-year, 2-year, and 3-year OS for the TCGA GBM and CGGA cohorts, and the predicted 2-year OS was approximated to the actual 2-year OS (Figures 7(b) and 7(e)). Kaplan-Meier curves showed a significant difference in OS between the high-risk and low-risk groups based on the clinical-featured risk model (Figures 7(c) and 7(f)). Moreover, ROC curve analysis was performed to estimate the predictive accuracy of the clinical-featured risk model. As shown in Figures 7(d) and 7(g), the AUC of ROC curves were 0.765 and 0.785 in the TCGA and CGGA cohorts, respectively, suggesting that the clinical-featured risk model had better accuracy and stability than the risk score model based on thrombosis-associated gene signature. In conclusion, the above results showed that the clinical-featured risk model had strong prognostic capabilities for GBM patients.

## 4. Discussion

Although significant advances have been made in surgical treatment, radiotherapy, and chemotherapy of GBM, the prognosis of GBM patients is still poor. Recent studies based on genetic and epigenetic biomarkers have improved the accuracy of prognosis prediction of GBM patients and optimized the treatment strategy of GBM [1, 28–33]. For example, *IDH* mutation, 1p/19q deletion, TP53, and ATRX were widely identified as the prognostic markers for GBM. Currently, thrombosis-associated genes have also been found to play vital roles in tumor progression, metastasis, tumor immunity, and therapeutic resistance. This study aimed to develop a prognostic model for GBM based on thrombosis-associated genes and ultimately help physicians estimate patient outcomes and design appropriate treatment strategies.

This study obtained a novel gene signature composed of thirteen thrombosis-associated genes by analyzing the TCGA GBM and the CGGA datasets. Based on the LASSO regression coefficients of 13 thrombosis-associated genes, we constructed a risk score model that can be used as a prognostic indicator for GBM patients in the TCGA and CGGA datasets. The signature can stratify patients according to their risk score. As results showed, high-risk patients have worsened survival in these datasets. We next comprehensively annotated the risk score's biological and immune-related functions. Several potential drug compounds related to the risk score were screened out. We finally determined the risk score as an independent prognostic factor in the nomogram incorporating other elements (age, gender, *IDH* mutation, radiotherapy, and chemotherapy). Among the 13 identified genes, CD59 has long been recognized as the complement membrane regulator of malignant GBM [34]. CFH, a circular RNA complement factor, has promoted GBM progression [35].

Similarly, hypoxia-induced LBH, a highly conserved transcription cofactor, participates in embryonic development and promotes tumor progression of GBM [36], and hypoxia upregulates the expression of PLAT in GBM cells [37]. Besides, FAP, overexpressed in several kinds of brain cancers, has been reported to be an excellent target for immunotherapy [38]. MASP-1, a significant mediator in the lectin complement signaling pathway of the innate immune response, has been overexpressed in GBM cell lines [39]. Moreover, a recent study revealed that PDGFA/PDGFR*α*-regulated GOLM1 critically enhances the tumor progression ability through activating AKT signaling in GBM [40].

Among the expression level and clinical correlation of thirteen candidate genes in patterns, *SERPING1*and *ANAX2* are of particular interest. SERPING1 (also known as C1-INH) is a plasma protein-encoding C1 inhibitor that plays a vital role in component regulation, coagulation, and fibrinolysis. *SERPING1* deficiency has been linked to the development of hereditary angioedema, sepsis, and pancreatic cancer. In line with previous studies, our study also found that the *SERPING1* gene is highly expressed in GBM and suggests a poor prognosis in patients with GBM. However, the exact role of SERPING1 in GBMs requires further investigation. ANXA2 is a member of the annexin family encoding a calcium-dependent phospholipid-binding protein that regulates cellular growth and signals transduction pathways. ANXA2 overexpression has been connected with various cancer growths, invasion, metastasis, and drug resistance. Previous evidence indicated that miR155HG was overexpressed and acts as a tumor suppressor to upregulate ANXA2 forming a loop that could promote the GBM's malignant progression. In this study, we found that the overexpression of *ANXA2* predicted a worse prognosis in GBM.

Given the vital roles of the 13 thrombosis-associated genes in GBM, a tumorigenic functional annotation is likely to be observed in the corresponding thrombosis-associated gene signature. As expected, the prognostic signature was enriched in Kappa B kinase NF-*κ*B signaling, the regulation of extrinsic apoptotic signaling pathway, the apoptosis, the focal adhesion, and the Toll-like receptor signaling pathway, the P53 pathway, and the TNF*α* signaling via NF-*κ*B, all of which were important signaling pathways regulating tumor proliferation and tumor progression. Notably, the prognostic signature was also associated with tumor metastasis. A close connection between m6A methylation regulators and the prognostic signature was observed, and the high score group had higher expression of writer WTAP. This also indicated the hazardous role of the prognostic signature in GBM patients.

Tumor microenvironment (TME) has been proposed to mediate the progression of GBMs critically [41–43]. The various immune infiltrating cells within the TME play an essential role in the crosstalk between GBM cells and TME [41–45]. In this study, multiple immune regulatory cells, including M2 macrophages and fibroblasts, were more expressed in the high-score group. The consistent findings proved that the thrombosis-associated gene signature was involved in an immunosuppressive microenvironment. Since GBMs are highly heterogeneous among individuals, we analyzed the characteristics of somatic mutations in the high-risk and low-risk groups. Our results proved that the tumor-suppressive gene, *TP53*, was mutated more frequently in the low-risk score group, while the oncogenic gene, *PTEN*, was mutated more commonly in the high-risk score group. This was by the previous finding, which further proved the prognostic value of the thrombosis-associated gene signature.

The current treatment modality for gliomas, especially GBM, is limited and dismal. To a large extent, novel therapeutic options would help promote the clinical management of GBM patients. In the analysis of potential drug compounds in risk score groups, all identified drugs from PRISM and CTRP have lower AUC values in the high-risk score group, suggesting that GBM patients in the high-risk group have increased sensitivity to the compounds-based therapy. PRISM-derived and CTRP-derived compounds were highly correlated with the thrombosis-associated signature. The above findings indicated that these compounds could be potentially applied in treating GBM patients. These identified compounds could be explored in a clinical trial for more robust verification of their therapeutic efficacy. Finally, a nomogram incorporating risk score, age, gender, *IDH* mutation, radiotherapy, and chemotherapy was constructed, which showed high sensitivity in predicting survival outcomes of GBM patients.

Although the new signature established by this research provides new biomarkers for thrombosis prevention in GBM patients and gives the foundation for developing anticoagulation therapy, there are still many deficiencies. For example, our risk score model is based on retrospective data and needs future research. Furthermore, apart from the two genes in our study, single genes' potential function and mechanism need to be further explored.

## 5. Conclusion

In sum, an independent prognostic risk score model based on the mRNA expression of 13 thrombosis-associated genes that were phenotypically significantly associated with immune invasion and somatic mutation in GBM was constructed using LASSO regression. We believe that the findings may provide a theoretical basis for the molecular diagnosis and individualized treatment for glioblastomas.

## Data Availability

All data used in this work can be acquired from The Cancer Genome Atlas (TCGA) (https://xenabrowser.net/) and the Chinese Glioma Genome Atlas (CGGA) (https://www.cgga.org.cn/) datasets.

## Supplementary Materials

Supplementary Materials Figure S1: heatmap depicting the expression difference of thirteen prognostic thrombosis-associated genes in TCGA training set, TCGA test set, TCGA sum set, and CGGA sum set. The column represents each patient and the row represents 13 genes' expression level. Figure S2: the thirteen prognostic thrombosis-associated genes have independent prognostic value in TCGA dataset and CGGA dataset. ((A)–(M)) Kaplan-Meier curves analysis correlation of OS among different risk groups and 13 genes. Figure S3: expression difference of *IDH* mutation status in the thirteen prognostic thrombosis-associated genes in (A) TCGA and (B) CGGA, respectively. Figure S4: functional enrichment analyses of thrombosis-associated gene signature in gliomas. (A) GO, KEGG, and HALLMARK analyses for thrombosis-associated prognostic genes in CGGA. (B) The expression of known signature in high-risk and low-risk groups in CGGA. (C) The expression of m6A-related genes in high-risk and low-risk groups in CGGA. GO, Gene ontology; KEGG, Kyoto Encyclopedia of Genes and Genomes; m6A, N6-methyladenosine. Figure S5: immunological function analysis of thrombosis-associated gene signature in gliomas. Heatmap shows the differential cellular immune responses between high-risk and low-risk groups analyzed by (A) ESTIMATE, MCP-counter, and TIMER algorithms and (B) xCell algorithm in CGGA. Figure S6: survival analysis of high-risk and low-risk groups with different clinicopathological factors and the prediction of chemotherapy response. (A) Kaplan-Meier survival curves of patients in the high-risk and low-risk groups with aged 65 years or older and those below 65 years of age in the CGGA glioma cohort. (B) Kaplan-Meier survival curves of patients in the high-risk and low-risk group with *IDH* mutation or wild-type *IDH* in CGGA glioma cohort. ((C) and (D)) Kaplan-Meier survival of patients in the high-risk and low-risk groups receiving radiotherapy and chemotherapy in the CGGA glioma cohort. Table S1: thrombosis-associated genes list. Table S2: Lasso regression coefficients of 13 thrombosis-associated genes.

## References

[1] Zhang H., Wang R., Yu Y., Liu J., Luo T., Fan F. (2019). Glioblastoma treatment modalities besides surgery. *Journal of Cancer*.

[2] Louis D. N., Perry A., Reifenberger G. (2016). The 2016 world health organization classification of tumors of the central nervous system: a summary. *Acta Neuropathologica*.

[3] Ricard D., Idbaih A., Ducray F., Lahutte M., Hoang-Xuan K., Delattre J.-Y. (2012). Primary brain tumours in adults. *The Lancet*.

[4] Huse J. T., Holland E. C. (2010). Targeting brain cancer: advances in the molecular pathology of malignant glioma and medulloblastoma. *Nature Reviews Cancer*.

[5] Yang K., Wu Z., Zhang H. (2022). Glioma targeted therapy: insight into future of molecular approaches. *Molecular Cancer*.

[6] Louis D. N., Perry A., Burger P. (2014). International society of neuropathology-Haarlem consensus guidelines for nervous system tumor classification and grading. *Brain Pathology*.

[7] Brennan C. W., Verhaak R. G., McKenna A. (2013). The somatic genomic landscape of glioblastoma. *Cell*.

[8] Thaler J., Ay C., Kaider A. (2014). Biomarkers predictive of venous thromboembolism in patients with newly diagnosed high-grade gliomas. *Neuro-Oncology*.

[9] Sciacca F. L., Ciusani E., Silvani A. (2004). Genetic and plasma markers of venous thromboembolism in patients with high grade glioma. *Clinical Cancer Research*.

[10] Hamada K., Kuratsu J.-I., Saitoh Y., Takeshima H., Nishi T., Ushio Y. (1996). Expression of tissue factor correlates with grade of malignancy in human glioma. *Cancer*.

[11] Saidak Z., Soudet S., Lottin M. (2021). A pan-cancer analysis of the human tumor coagulome and its link to the tumor immune microenvironment. *Cancer Immunology, Immunotherapy*.

[12] Rong Y., Post D. E., Pieper R. O., Durden D. L., Van Meir E. G., Brat D. J. (2005). PTEN and hypoxia regulate tissue factor expression and plasma coagulation by glioblastoma. *Cancer Research*.

[13] Rong Y., Belozerov V. E., Tucker-Burden C. (2009). Epidermal growth factor receptor and PTEN modulate tissue factor expression in glioblastoma through JunD/activator protein-1 transcriptional activity. *Cancer Research*.

[14] Goeman J. J. (2010). L1 penalized estimation in the Cox proportional hazards model. *Biometrical Journal. Biometrische Zeitschrift*.

[15] Zhang Y., Li H., Zhang W., Che Y., Bai W., Huang G. (2018). LASSO based CoxPH model identifies an 11lncRNA signature for prognosis prediction in gastric cancer. *Molecular Medicine Reports*.

[16] Yoshihara K., Shahmoradgoli M., Martínez E. (2013). Inferring tumour purity and stromal and immune cell admixture from expression data. *Nature Communications*.

[17] Becht E., Giraldo N. A., Lacroix L. (2016). Estimating the population abundance of tissue-infiltrating immune and stromal cell populations using gene expression. *Genome Biology*.

[18] Aran D., Hu Z., Butte A. J. (2017). xCell: digitally portraying the tissue cellular heterogeneity landscape. *Genome Biology*.

[19] Li T., Fan J., Wang B. (2017). TIMER: a web server for comprehensive analysis of tumor-infiltrating immune cells. *Cancer Research*.

[20] Koboldt D. C., Zhang Q., Larson D. E. (2012). VarScan 2: somatic mutation and copy number alteration discovery in cancer by exome sequencing. *Genome Research*.

[21] Leiserson M. D., Wu H.-T., Vandin F., Raphael B. J. (2015). CoMEt: a statistical approach to identify combinations of mutually exclusive alterations in cancer. *Genome Biology*.

[22] Geeleher P., Cox N., Huang R. S. (2014). pRRophetic: an R package for prediction of clinical chemotherapeutic response from tumor gene expression levels. *PLoS One*.

[23] Robin X., Turck N., Hainard A. (2011). pROC: an open-source package for R and S+ to analyze and compare ROC curves. *BMC Bioinformatics*.

[24] Núñez F. J., Mendez F. M., Kadiyala P. (2019). IDH1-R132H acts as a tumor suppressor in glioma via epigenetic up-regulation of the DNA damage response. *Science Translational Medicine*.

[25] Chang K., Wang G., Lou J. (2020). lncRNA TTN‑AS1 upregulates RUNX1 to enhance glioma progression via sponging miR‑27b‑3p. *Oncology Reports*.

[26] Chen P., Zhao D., Li J. (2019). Symbiotic macrophage-glioma cell interactions reveal synthetic lethality in PTEN-null glioma. *Cancer Cell*.

[27] Eskilsson E., Røsland G. V., Solecki G. (2018). EGFR heterogeneity and implications for therapeutic intervention in glioblastoma. *Neuro-Oncology*.

[28] Zhang H., Zhou Y., Cui B., Liu Z., Shen H. (2020). Novel insights into astrocyte-mediated signaling of proliferation, invasion and tumor immune microenvironment in glioblastoma. *Biomedicine & Pharmacotherapy*.

[29] Zhang H., Chen Z., Wang Z. (2021). Correlation between APOBEC3B expression and clinical characterization in lower-grade gliomas. *Frontiers in Oncology*.

[30] Zhang H., Cui B., Zhou Y. (2021). B2M overexpression correlates with malignancy and immune signatures in human gliomas. *Scientific Reports*.

[31] Zhang H., Fan F., Yu Y. (2020). Clinical characterization, genetic profiling, and immune infiltration of TOX in diffuse gliomas. *Journal of Translational Medicine*.

[32] Zhang H., He J., Dai Z. (2021). PDIA5 is correlated with immune infiltration and predicts poor prognosis in gliomas. *Frontiers in Immunology*.

[33] Zhang H., Zhou Y., Cheng Q. (2020). PDIA3 correlates with clinical malignant features and immune signature in human gliomas. *Aging*.

[34] Mäenpää A., Junnikkala S., Hakulinen J., Timonen T., Meri S. (1996). Expression of complement membrane regulators membrane cofactor protein (CD46), decay accelerating factor (CD55), and protectin (CD59) in human malignant gliomas. *American Journal of Pathology*.

[35] Bian A., Wang Y., Liu J. (2018). Circular RNA complement factor H (CFH) promotes glioma progression by sponging miR-149 and regulating AKT1. *Medical Science Monitor*.

[36] Jiang Y., Zhou J., Zou D. (2019). Overexpression of limb-bud and heart (LBH) promotes angiogenesis in human glioma via VEGFA-mediated ERK signalling under hypoxia. *EBioMedicine*.

[37] Minchenko O. H., Kharkova A. P., Kubaichuk K. I., Minchenko D. O., Hlushchak N. A., Kovalevska O. V. (2014). Effect of hypoxia on the expression of CCN2, PLAU, PLAUR, SLURP1, PLAT and ITGB1 genes in ERN1 knockdown U87 glioma cells. *Ukrainian Biochemical Journal*.

[38] Ebert L. M., Yu W., Gargett T. (2020). Endothelial, pericyte and tumor cell expression in glioblastoma identifies fibroblast activation protein (FAP) as an excellent target for immunotherapy. *Clinical & Translational Immunology*.

[39] Pagliara V., Parafati M., Adornetto A. (2018). Dibutyryl cAMP- or Interleukin-6-induced astrocytic differentiation enhances mannose binding lectin (MBL)-associated serine protease (MASP)-1/3 expression in C6 glioma cells. *Archives of Biochemistry and Biophysics*.

[40] Xu R., Ji J., Zhang X. (2017). PDGFA/PDGFR*α*-regulated GOLM1 promotes human glioma progression through activation of AKT. *Journal of Experimental & Clinical Cancer Research*.

[41] Zhang H., Wang Z., Dai Z. (2021). Novel immune infiltrating cell signature based on cell pair algorithm is a prognostic marker in cancer. *Frontiers in Immunology*.

[42] Zhang N., Zhang H., Wang Z. (2021). Immune infiltrating cells-derived risk signature based on large-scale analysis defines immune landscape and predicts immunotherapy responses in glioma tumor microenvironment. *Frontiers in Immunology*.

[43] Zhang H., Luo Y.-B., Wu W. (2021). The molecular feature of macrophages in tumor immune microenvironment of glioma patients. *Computational and Structural Biotechnology Journal*.

[44] Wang Z., Su G., Dai Z. (2021). Circadian clock genes promote glioma progression by affecting tumour immune infiltration and tumour cell proliferation. *Cell Proliferation*.

[45] Zhang H., Dai Z., Wu W. (2021). Regulatory mechanisms of immune checkpoints PD-L1 and CTLA-4 in cancer. *Journal of Experimental & Clinical Cancer Research*.

