# Supplementary material for: A Novel Thrombosis-Related Signature for Predicting Survival and Drug Compounds in Glioblastoma
Source: J Oncol. 2022 Jul 13;2022:6792850. doi: 10.1155/2022/6792850 (PMC9300384; doi:10.1155/2022/6792850)
Supplement: Supplementary Materials — Figure S1: heatmap depicting the expression difference of thirteen prognostic thrombosis-associated genes in TCGA training set, TCGA test set, TCGA sum set, and CGGA sum set. The column represents each patient and the row represents 13 genes' expression level. Figure S2: the thirteen prognostic thrombosis-associated genes have independent prognostic value in TCGA dataset and CGGA dataset. ((A)–(M)) Kaplan-Meier curves analysis correlation of OS among different risk groups and 13 genes. Figure S3: expression difference of IDH mutation status in the thirteen prognostic thrombosis-associated genes in (A) TCGA and (B) CGGA, respectively. Figure S4: functional enrichment analyses of thrombosis-associated gene signature in gliomas. (A) GO, KEGG, and HALLMARK analyses for thrombosis-associated prognostic genes in CGGA. (B) The expression of known signature in high-risk and low-risk groups in CGGA. (C) The expression of m6A-related genes in high-risk and low-risk groups in CGGA. GO, Gene ontology; KEGG, Kyoto Encyclopedia of Genes and Genomes; m6A, N6-methyladenosine. Figure S5: immunological function analysis of thrombosis-associated gene signature in gliomas. Heatmap shows the differential cellular immune responses between high-risk and low-risk groups analyzed by (A) ESTIMATE, MCP-counter, and TIMER algorithms and (B) xCell algorithm in CGGA. Figure S6: survival analysis of high-risk and low-risk groups with different clinicopathological factors and the prediction of chemotherapy response. (A) Kaplan-Meier survival curves of patients in the high-risk and low-risk groups with aged 65 years or older and those below 65 years of age in the CGGA glioma cohort. (B) Kaplan-Meier survival curves of patients in the high-risk and low-risk group with IDH mutation or wild-type IDH in CGGA glioma cohort. ((C) and (D)) Kaplan-Meier survival of patients in the high-risk and low-risk groups receiving radiotherapy and chemotherapy in the CGGA glioma cohort. Table S1: thrombosis-associate [file 6792850.f1.zip › 6792850.f1/FigS5.pdf]

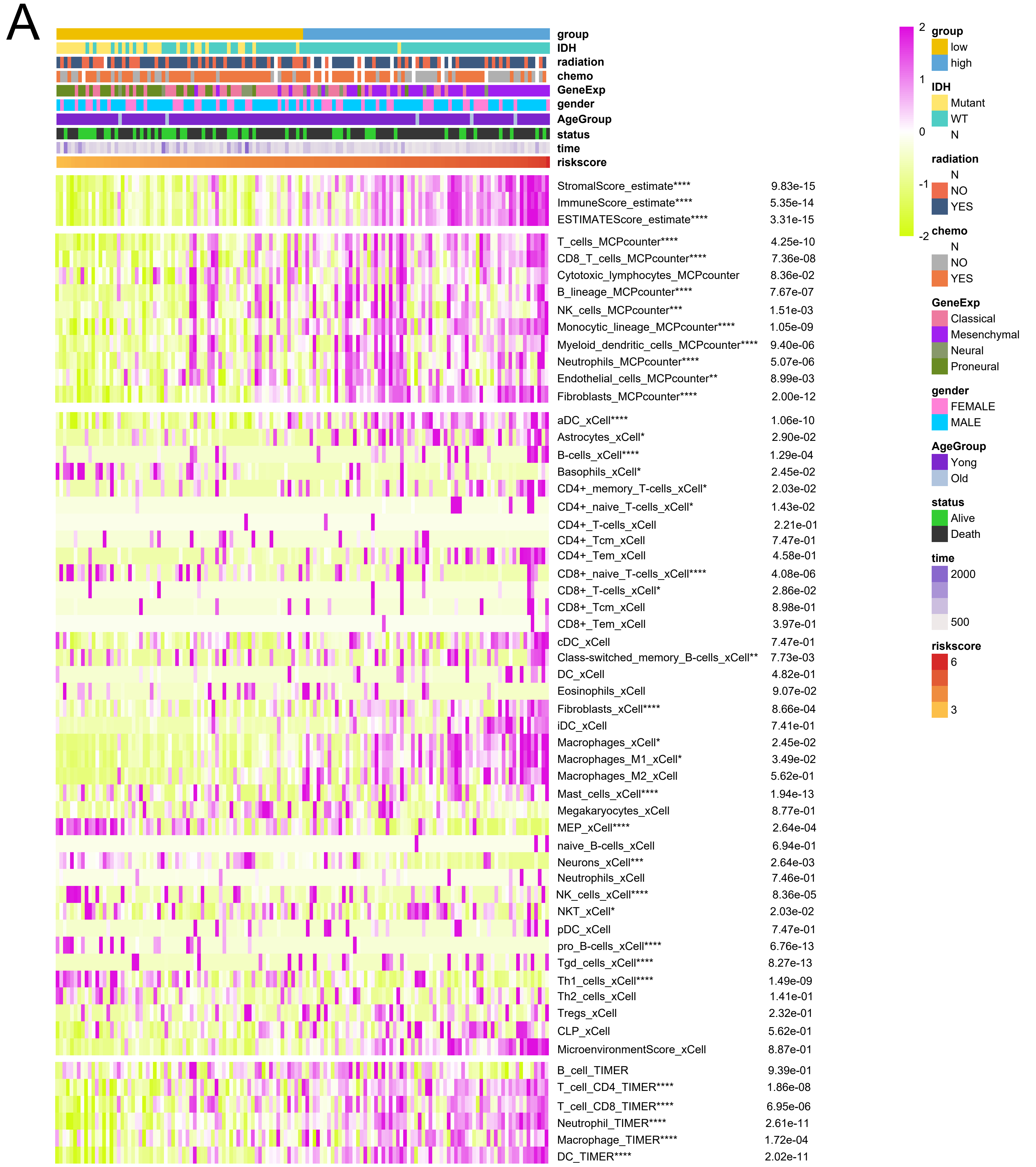

A
group
IDH
radiation
chemo
GeneExp
gender
AgeGroup
status
time
riskscore
StromalScore_estimate**** 9.83e-15
ImmuneScore_estimate**** 5.35e-14
ESTIMATEScore_estimate**** 3.31e-15
T_cells_MCPcounter**** 4.25e-10
CD8_T_cells_MCPcounter**** 7.36e-08
Cytotoxic_lymphocytes_MCPcounter 8.36e-02
B_lineage_MCPcounter**** 7.67e-07
NK_cells_MCPcounter*** 1.51e-03
Monocytic_lineage_MCPcounter**** 1.05e-09
Myeloid_dendritic_cells_MCPcounter**** 9.40e-06
Neutrophils_MCPcounter**** 5.07e-06
Endothelial_cells_MCPcounter** 8.99e-03
Fibroblasts_MCPcounter**** 2.00e-12
aDC_xCell**** 1.06e-10
Astrocytes_xCell* 2.90e-02
B-cells_xCell**** 1.29e-04
Basophils_xCell* 2.45e-02
CD4+_memory_T-cells_xCell* 2.03e-02
CD4+_naive_T-cells_xCell* 1.43e-02
CD4+_T-cells_xCell 2.21e-01
CD4+_Tcm_xCell 7.47e-01
CD4+_Tem_xCell 4.58e-01
CD8+_naive_T-cells_xCell**** 4.08e-06
CD8+_T-cells_xCell* 2.86e-02
CD8+_Tcm_xCell 8.98e-01
CD8+_Tem_xCell 3.97e-01
cDC_xCell 7.47e-01
Class-switched_memory_B-cells_xCell** 7.73e-03
DC_xCell 4.82e-01
Eosinophils_xCell 9.07e-02
Fibroblasts_xCell**** 8.66e-04
iDC_xCell 7.41e-01
Macrophages_xCell* 2.45e-02
Macrophages_M1_xCell* 3.49e-02
Macrophages_M2_xCell 5.62e-01
Mast_cells_xCell**** 1.94e-13
Megakaryocytes_xCell 8.77e-01
MEP_xCell**** 2.64e-04
naive_B-cells_xCell 6.94e-01
Neurons_xCell*** 2.64e-03
Neutrophils_xCell 7.46e-01
NK_cells_xCell**** 8.36e-05
NKT_xCell* 2.03e-02
pDC_xCell 7.47e-01
pro_B-cells_xCell**** 6.76e-13
Tgd_cells_xCell**** 8.27e-13
Th1_cells_xCell**** 1.49e-09
Th2_cells_xCell 1.41e-01
Tregs_xCell 2.32e-01
CLP_xCell 5.62e-01
MicroenvironmentScore_xCell 8.87e-01
B_cell_TIMER 9.39e-01
T_cell_CD4_TIMER**** 1.86e-08
T_cell_CD8_TIMER**** 6.95e-06
Neutrophil_TIMER**** 2.61e-11
Macrophage_TIMER**** 1.72e-04
DC_TIMER**** 2.02e-11
2
1
0
-1
-2
group
low
high
IDH
Mutant
WT
N
radiation
N
NO
YES
chemo
N
NO
YES
GeneExp
Classical
Mesenchymal
Neural
Proneural
gender
FEMALE
MALE
AgeGroup
Yong
Old
status
Alive
Death
time
2000
500
riskscore
6
3
