# Supplementary material for: A Novel Thrombosis-Related Signature for Predicting Survival and Drug Compounds in Glioblastoma
Source: J Oncol. 2022 Jul 13;2022:6792850. doi: 10.1155/2022/6792850 (PMC9300384; doi:10.1155/2022/6792850)
Supplement: Supplementary Materials — Figure S1: heatmap depicting the expression difference of thirteen prognostic thrombosis-associated genes in TCGA training set, TCGA test set, TCGA sum set, and CGGA sum set. The column represents each patient and the row represents 13 genes' expression level. Figure S2: the thirteen prognostic thrombosis-associated genes have independent prognostic value in TCGA dataset and CGGA dataset. ((A)–(M)) Kaplan-Meier curves analysis correlation of OS among different risk groups and 13 genes. Figure S3: expression difference of IDH mutation status in the thirteen prognostic thrombosis-associated genes in (A) TCGA and (B) CGGA, respectively. Figure S4: functional enrichment analyses of thrombosis-associated gene signature in gliomas. (A) GO, KEGG, and HALLMARK analyses for thrombosis-associated prognostic genes in CGGA. (B) The expression of known signature in high-risk and low-risk groups in CGGA. (C) The expression of m6A-related genes in high-risk and low-risk groups in CGGA. GO, Gene ontology; KEGG, Kyoto Encyclopedia of Genes and Genomes; m6A, N6-methyladenosine. Figure S5: immunological function analysis of thrombosis-associated gene signature in gliomas. Heatmap shows the differential cellular immune responses between high-risk and low-risk groups analyzed by (A) ESTIMATE, MCP-counter, and TIMER algorithms and (B) xCell algorithm in CGGA. Figure S6: survival analysis of high-risk and low-risk groups with different clinicopathological factors and the prediction of chemotherapy response. (A) Kaplan-Meier survival curves of patients in the high-risk and low-risk groups with aged 65 years or older and those below 65 years of age in the CGGA glioma cohort. (B) Kaplan-Meier survival curves of patients in the high-risk and low-risk group with IDH mutation or wild-type IDH in CGGA glioma cohort. ((C) and (D)) Kaplan-Meier survival of patients in the high-risk and low-risk groups receiving radiotherapy and chemotherapy in the CGGA glioma cohort. Table S1: thrombosis-associate [file 6792850.f1.zip › 6792850.f1/Table S1 (1).pdf]

Table S1. Thrombosis associated gene list.
A2M
ABAT
ACCN1
ADAMTS18
ADRB2
ALOX12
ANO6
ANXA2
ANXA5
APOE
APOH
BDKRB1
BDKRB2
C1QA
C1QB
C1QBP
C1QC
C1QTNF1
C1R
C1S
C2
C3
C3AR1
C4A
C4B
C4BPA
C4BPB
C5
C5AR1
C6
C7
C8A
C8B
C8G
C9
CAV1
CD34
CD36
CD46
CD55
CD59
CFB
CFD
CFH
CFI
CLEC4M
CPB2
CR1
CR2
EDN1
ENPP4
EPB49
F10
F11
F12
F13A1
F13B

F2
F2R
F2RL1
F3
F5
F7
F8
F9
FAM46A
FAP
FBLN1
FCER1G
FGA
FGB
FGG
FOXA2
GP1BA
GP1BB
GP5
GP9
HPSE
HRG
HS3ST5
KLKB1
KNG1
KRT1
LBH
LYN
MASP1
MASP2
MBL2
NFE2L2
NOS3
PDGFA
PDGFB
PDGFRA
PLAT
PLAU
PLAUR
PLEK
PLG
PRCP
PRKCA
PRKCD
PRKCQ
PRKG1
PROC
PROCR
PROS1
PSEN1
PTGER3
S100A9
SCARA5
SELP
SERPINA1
SERPINA5
SERPINB2
SERPINC1

SERPIND1
SERPINE1
SERPINE2
SERPINF2
SERPING1
STAB2
STX2
STXBP5
SYK
TC2N
TEC
TFPI
THBD
THBS1
TLR4
TMPRSS6
TSPAN8
TXK
UBASH3B
USF1
VAV2
VPS33B
VTN
VWF
ZAP70
